# Landelijk gezondheidsonderzoek bij rampen: Achtergrond en opzet van de meerjarige integrale Gezondheidsmonitor COVID-19

**DOI:** 10.1007/s12508-021-00326-7

**Published:** 2021-12-20

**Authors:** Mark Bosmans, Elske Marra, Nannah Tak, Noortje Jansen, Femke de Zwart, Michel Dückers

**Affiliations:** 1 Nivel – Nederlands instituut voor onderzoek van de gezondheidszorg, Utrecht, Nederland; 2 Rijksinstituut voor Volksgezondheid en Milieu, Bilthoven, Nederland; 3 GGD GHOR Nederland, Utrecht, Nederland; 4 ARQ Nationaal Psychotrauma Centrum, Diemen, Nederland; 5 Faculteit Gedrags- & Maatschappijwetenschappen, Rijksuniversiteit Groningen, Groningen, Nederland

## Abstract

De coronacrisis en de gevolgen die deze op de gezondheid van de Nederlandse bevolking heeft gaan de normale regionale onderzoeksaanpak te boven. Daarom heeft het Netwerk GOR-COVID-19 – dat bestaat uit GGD GHOR Nederland (namens de GGD’en), RIVM, Nivel en ARQ Nationaal Psychotrauma Centrum – het initiatief genomen voor een landelijk onderzoeksprogramma om de impact van de coronapandemie op de mentale en fysieke gezondheid van de Nederlandse bevolking op lange termijn te monitoren: de integrale Gezondheidsmonitor COVID-19. In dit artikel beschrijven we de achtergrond en opzet van deze gezondheidsmonitor, die erop is gericht onderzoeksbevindingen toepasbaar te maken voor praktijk en beleid, zowel lokaal als nationaal.

## Abstract (en)

The consequences of the COVID-19 pandemic for the health and wellbeing of the Dutch population exceed the normal regional research-approach. That is why the ‘network GOR-COVID-19’ – comprised of GGD GHOR Nederland (representing the municipal health services), RIVM, Nivel and ARQ National Psychotrauma Centre – has taken the initiative for a national research program to monitor the health impact of the corona crisis over time. In this article we describe the background and design of a comprehensive longitudinal health monitor that combines and harmonizes multiple data sources in order to provide perspectives for practice and policy, on both a local and national level.

## Bovenregionale samenwerking: het Netwerk GOR-COVID-19

Bij de organisatie van crisisbeheersing bij rampen en andere crises wordt uitgegaan van lokale capaciteit en regie. Als het gaat om het volgen van de gezondheidsgevolgen en risico’s over de tijd, ligt dit niet anders. ‘Gezondheidsonderzoek bij rampen’ of kortweg ‘GOR’ is geënt op het verkrijgen van goed inzicht in gezondheid(srisico’s) en veranderingen die optreden in de grillige tijdlijn van een ramp. Deze informatie is onmisbaar voor beleidsmakers en professionals. De publieke gezondheidsorganisatie, inclusief GOR, is in Nederland regionaal georganiseerd. De huidige crisis vergt echter een bovenregionale aanpak, ook als het gaat om het onderzoeken van de gezondheidsgevolgen. Daarom heeft het Netwerk GOR-COVID-19 het initiatief genomen tot een landelijk monitoringsprogramma om de gezondheidsgevolgen (mentaal en fysiek) van de coronacrisis op een geharmoniseerde wijze te volgen. Het Netwerk GOR-COVID-19 bestaat uit GGD GHOR Nederland (namens de GGD’en), RIVM, Nivel en ARQ Nationaal Psychotrauma Centrum. De ‘integrale Gezondheidsmonitor COVID-19’ – GOR op lokaal, regionaal en landelijk niveau, waarbij verschillende typen databronnen bijeengebracht worden – heeft een looptijd van vijf jaar (2021–2025). Het project wordt gefinancierd door het ministerie van VWS en krijgt subsidie van ZonMw. In deze bijdrage beschrijven we de doelstellingen, achtergrond en opzet van deze gezondheidsmonitor.

## Coronacrisis

 De wereldwijde COVID-19-pandemie houdt sinds maart 2020 ook Nederland in zijn greep. De pandemie heeft een groot beroep gedaan op de zorgcapaciteit en resulteerde in een serie maatregelen gericht op beheersing van de uitbraak. De samenleving ging op slot: men bleef thuis, hield afstand en bijna alle openbare gelegenheden – inclusief scholen – werden een tijd lang gesloten. Op het moment van schrijven (oktober 2021) heeft de pandemie in Nederland geleid tot bijna 1,8 miljoen bevestigde besmettingen, en ruim 18,000 geregistreerde dodelijke coronaslachtoffers [1]. Waarschijnlijk liggen deze cijfers in werkelijkheid hoger, omdat niet iedereen getest is en niet ieder overlijden als gevolg van het coronavirus als zodanig bij de GGD gemeld wordt.

Naast geïnfecteerde personen zijn ook veel mensen indirect getroffen door de coronacrisis – de coronacrisis raakt de samenleving als geheel. De directe en indirecte gevolgen van het virus en de maatregelen die genomen worden om verspreiding van het virus te voorkomen vormen een mogelijke bedreiging voor de gezondheid en het welzijn van de bevolking op zowel korte als lange termijn.

Allerlei gezondheidseffecten van infectie(s) met het COVID-19 virus en maatregelen die zijn genomen zijn direct waarneembaar. In de eerste plaats geldt dit voor mensen die na infectie milde of ernstige klachten ontwikkelen, soms zo ernstig dat een ziekenhuisopname of zelfs overlijden volgt. Een ander deel van deze mensen houdt na besmetting langdurige lichamelijke klachten en vermoeidheid. Ook zijn er mensen die met serieuze – niet aan COVID-19 gerelateerde – klachten de weg naar zorg niet of later hebben gevonden, omdat de zorg zich een periode primair heeft gericht op COVID-19-patiënten. Daarnaast zijn er mensen die kampen met mentale problemen veroorzaakt of bestendigd door de pandemie [2, 3]. Uiteindelijk is de gehele bevolking, ook degenen zonder directe zorgvraag, geraakt door de genomen maatregelen. Bovendien verwachten experts negatieve gezondheidseffecten van chronische stress en onzekerheid door de duur van de crisis, al dan niet versterkt door overheidsmaatregelen. Deze negatieve effecten kunnen bovendien toenemen door toekomstige ontwikkelingen – nieuwe besmettingsgolven, verlies van werk en inkomen, economische recessie, sociale deprivatie, minder beweging, verschraling van het maatschappelijk leven of onvrede over het overheidsbeleid [4–6]. Het is voorstelbaar dat deze effecten specifieke groepen burgers die al kwetsbaar waren extra hard raken. Dit kan leiden tot een versterking van sociaaleconomische gezondheidsverschillen [7].

## Gezondheidsonderzoek bij rampen

Om de gezondheidseffecten van deze crisis op verschillende groepen in verschillende fasen zoveel mogelijk te beperken is informatie nodig voor beleid en praktijk. Een goede beeld- en oordeelsvorming over welke zorg nodig is en waar versterking van de zorgcapaciteit en -organisatie gewenst is kan hier richting aan geven [8]. Hiervoor zijn inzicht in en overzicht van de gezondheidseffecten in brede zin onmisbaar. Die gedachte ligt ten grondslag aan de eerder beschreven crisistaak GOR. GOR, een wettelijke taak beschreven in de Wet publieke gezondheid (Wpg, artikel 2, lid 1 Besluit publieke gezondheid), omvat onderzoek naar de psychische en fysieke gezondheid en naar zorg- en ondersteuningsbehoeften na een crisis of ramp.

GOR kan op verschillende manieren bijdragen aan de gezondheid van getroffenen. In hoofdlijnen zijn vier verschillende doelen te onderscheiden[8, 9]:

- Zorginhoudelijk doel: bijdragen aan het goed afstemmen van zorg en behandeling van betrokkenen;
- Beleidsmatig/organisatorisch doel: bijdragen aan het afstemmen van beleid en maatregelen om in te spelen op de zorg- en ondersteuningsbehoeften van betrokkenen;
- Maatschappelijk doel: het afgeven van een signaal dat de gevolgen van de ramp of crisis goed in kaart worden gebracht;
- Wetenschappelijk doel: het bijdragen aan de kennisbasis omtrent de gevolgen van rampen en crises voor volgende gebeurtenissen.
- Het wetenschappelijk doel is nooit doorslaggevend bij de beslissing tot het inzetten van GOR, maar is wel belangrijk voor kennisopbouw in Nederland.

Tot nu toe is de ervaring met GOR in Nederland voornamelijk beperkt tot relatief kleinschalige, gelokaliseerde rampen. Het ging vrijwel altijd om ‘flitsrampen’, plotselinge gebeurtenissen met een onmiddellijke nasleep. Zo is gezondheidsonderzoek gestart na de vuurwerkramp in Enschede (2000), de Nieuwjaarsbrand in Volendam (2001), de poldercrash (2009) en de ramp met vlucht MH17 (2014). Het enige voorbeeld van longitudinaal GOR bij een langslepende crisis in Nederland betreft de aardbevingsproblematiek in Groningen [10].

Vanuit de Wpg zijn de gemeenten verantwoordelijk voor het uitvoeren van GOR, in de praktijk is deze taak belegd bij de regionale GGD. De GGD adviseert in de gebruikelijke gang van zaken het bevoegd gezag (meestal de gemeente) – eventueel met ondersteuning van de expertgroep Nazorg van het centrum Gezondheid en Milieu van het RIVM – over het wel of niet uitvoeren van GOR. Na een beslissing door het bevoegd gezag om GOR uit te voeren heeft de GGD een coördinerende rol bij het opzetten en uitvoeren van het onderzoek. Zoals gezegd vergt de huidige crisis vanwege de ingrijpende en landelijke aard van de impact echter een bovenregionale aanpak.

### Doelstellingen van de integrale Gezondheidsmonitor COVID-19

De hoofddoelstelling van de integrale Gezondheidsmonitor COVID-19 is het bieden van een goede informatiebasis wat betreft de fysieke en mentale gezondheidseffecten van de COVID-19-crisis, om lokale en regionale bestuurders te kunnen adviseren en ondersteunen bij beleidsvorming, en het aanreiken van handelingsperspectieven.

Subdoelstellingen zijn:

- het verwerven van inzicht in de directe effecten van het coronavirus (COVID-19) op de gezondheid;
- het verwerven van inzicht in de effecten van de maatregelen gericht op de beheersing van de pandemie op de gezondheid;
- het bevorderen van de doorgeleiding van kennis en inzichten naar landelijke, regionale en lokale beleidmakers en zorgverleners.

### Link met praktijk en beleid

De gezondheidsmonitor draagt bij aan het verkrijgen en doorgeleiden naar praktijk en beleid van inzicht in de effecten van de coronacrisis op de gezondheidssituatie van de bevolking. Om dit te optimaliseren hebben multidisciplinaire werkgroepen de taak om telkens wanneer nieuwe resultaten beschikbaar deze te duiden. Hieraan nemen vertegenwoordigers deel van de overheid, betrokken uitvoerende zorginstanties, het sociaal domein en instituten die een brug slaan tussen wetenschap, beleid en uitvoering. In de werkgroepen wordt een afweging gemaakt van bruikbaarheid van resultaten voor beleid en worden deze vertaald in concrete handelingsperspectieven. Ook op GGD-regioniveau worden dergelijke werkgroepen georganiseerd, om de vertaalslag te maken naar handelingsperspectieven voor lokale beleidsmakers en organisaties.

### Focus op gezondheid, en risico- en beschermende factoren

Steeds staan dezelfde gezondheidsmaten centraal. Het gaat om klassieke gezondheidsmaten in GOR, zoals stemmingsklachten en depressies, angsten, suïcidaliteit, middelengebruik, sociale en relationele problemen. Daarnaast worden ook non-specifieke klachten, chronische gezondheidsklachten en veranderingen in zorggebruik (inclusief medicatie) meegenomen. De klassieke GOR-gezondheidsmaten worden verder uitgebreid met andere gezondheidsthema’s binnen de publieke gezondheid, zoals eenzaamheid, bewegen en regie over het eigen leven. Uiteraard wordt voortgebouwd op een brede kennisbasis van risico- en beschermende factoren.

## Opzet van de integrale Gezondheidsmonitor COVID-19

### Opschaling vanuit reguliere structuren

In de gezondheidsmonitor wordt waar mogelijk voortgebouwd op de bestaande regionale en landelijke onderzoeksinfrastructuur vanuit GGD’en (zoals de gezondheidsmonitor jeugd en de gezondheidsmonitor volwassenen en ouderen), RIVM en Nivel. Deze reguliere structuur vormt – in opgeschaalde vorm – de basis voor de gezondheidsmonitor en levert cijfers op lokaal, regionaal en nationaal niveau, en maakt vergelijken mogelijk tussen gebieden en over de tijd, ook met de periode voorafgaand aan de uitbraak van het coronavirus in Nederland. Figuur 1 laat zien wanneer de verschillende onderdelen van de gezondheidsmonitor plaatsvinden.

**Figure 1:**
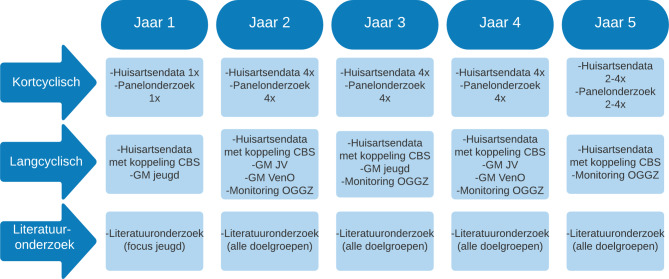

Wanneer gewenst of noodzakelijk – zoals bij het opzetten van de Openbare geestelijke gezondheidszorg (OGGZ)-monitor – wordt samengewerkt met andere afdelingen van het RIVM (zoals de afdeling Volksgezondheid en Zorg), kennisinstituten (zoals het Trimbos-instituut of Pharos) of andere onderzoeksgroepen (zoals biobank *Lifelines*). Ook wordt samenwerking gezocht met partijen die relevante doelgroepen volgen, bijvoorbeeld organisaties die betrokken zijn bij de hulpverlening aan OGGZ-doelgroepen.

Voor de gezondheidsmonitor is gekozen voor twee vormen van monitoring, die min of meer tegelijk ontwikkeld en uitgevoerd worden: kortcyclische en langcyclische monitoring. Deze combinatie biedt inzicht in patronen en ontwikkelingen in gezondheid over de tijd en in de factoren die daarbij van invloed zijn, maar biedt ook de mogelijkheid om op basis van nieuw verkregen inzicht (snel) te schakelen tussen beleidsniveaus en op te schalen. Bij beide typen monitoring worden nieuwe data verzameld via vragenlijsten en wordt gebruikgemaakt van routinematig verkregen zorgregistratiedata, afkomstig van huisartsenpraktijken (Nivel Zorgregistraties Eerste Lijn, NZR). Beide leveren andersoortige informatie op:

- Kortcyclische monitoring geeft inzicht in de mate waarin fysieke en mentale gezondheidsproblemen (op dit moment) voorkomen binnen de populatie.
- Langcyclische monitoring geeft inzicht in het verloop van klachten en zorggebruik over de tijd, en factoren die daarbij een rol spelen, met oog voor risicogroepen en regionale/lokale verschillen of andere opvallende zaken.

### Kortcyclische monitoring

Kortcyclische monitoring levert ongeveer vier keer per jaar een beperkte verzameling van geïnterpreteerde data op. De hoge frequentie van verschijnen draagt zorg voor inzicht in de actuele situatie. De data komen uit de NZR. Daarnaast worden zowel onder jeugd en jongvolwassenen samen (tot en met 25 jaar), als onder volwassenen door een extern onderzoeksbureau in opdracht van het netwerk metingen uitgezet in bestaande of nieuw op te richten representatieve panels.

### Langcyclische monitoring

Aan de basis van langcyclische monitoring liggen primaire data. Alle GGD’en in Nederland zijn hierbij betrokken. Zij zetten vragenlijsten uit voor verschillende leeftijdsgroepen: jeugd (klas 2 en 4 van het voortgezet onderwijs), jongvolwassenen en volwassenen en ouderen binnen hun bestaande panelstructuur. Voor de groep mensen die tot de OGGZ-doelgroep behoren wordt een nieuwe monitor ontwikkeld. Daarnaast worden data uit de NZR gekoppeld met CBS-data. Jaarlijks komen resultaten beschikbaar.

Langcyclische monitoring levert resultaten op gemeenteniveau (voor grotere gemeenten op wijk-/buurtniveau) ten behoeve van lokaal beleid. Waar nodig worden deze aangevuld met registraties vanuit RIVM of externe onderzoeksgroepen. De langcyclische monitoring biedt meer diepgang door analyses van trends en patronen, vergelijkbaarheid op alle verschillende beleidsniveaus en koppeling van meerdere gegevensbronnen.

### Inventarisatie, onderzoeksagenda en open subsidieoproep

Naast de monitoring wordt binnen het project jaarlijks een inventarisatie gehouden van lopende en afgeronde onderzoeken in Nederland met betrekking tot de gezondheidsgevolgen van de coronapandemie, en van internationale onderzoeksresultaten. De inzichten die hieruit voortkomen kunnen worden ingezet om de lang- en kortcyclische monitoring te optimaliseren, bijvoorbeeld als het gaat om het identificeren van groepen voor wie het risico op negatieve gezondheidsuitkomsten groter is. Daarnaast biedt deze inventarisatie een overzicht van kennishiaten en daarmee input voor de onderzoeksagenda voor verdiepend onderzoek. Tijdens de looptijd van de integrale Gezondheidsmonitor COVID-19 wordt twee keer een open subsidieoproep geplaatst (in 2022 en 2024) voor verdiepende onderzoeken die gebruikmaken van de verzamelde data.

## Coronacrisis als een kans voor versterking van de reguliere gezondheidsmonitoring

Crisis is kans. Het is een gevleugelde uitspraak. In dit artikel hebben we toegelicht hoe COVID-19 een acute aanleiding vormde om een brede monitoringsaanpak in te richten, die het zowel regionaal als landelijk mogelijk maakt om te anticiperen op gezondheidsrisico’s en ontwikkelingen daarin. Dat betekent dat de komende jaren op verschillende niveaus steeds monitoringsresultaten worden ingebracht in het gesprek tussen wetenschappers, professionals en beleidsmakers (de eerste rapportages gericht op de jeugd worden op dit moment – eind 2021 – opgesteld).

COVID-19 vormde een aanleiding, maar het ligt voor de hand dat de extra instrumenten, metingen en netwerken die tijdens de uitvoering van deze brede gezondheidsmonitor ontstaan, niet alleen ten goede zullen komen aan de GOR-taak in de toekomst, maar ook aan de reguliere gezondheidsmonitoring. Ook deze crisis legt immers bestaande gezondheidsrisico’s en kwetsbaarheden bloot die ook los van deze crisis aandacht verdienen van beleidsmakers en professionals.
